# Surgical treatment options for articular cartilage defects of the glenohumeral joint: A systematic review

**DOI:** 10.1177/17585732221142610

**Published:** 2022-12-14

**Authors:** Danielle Dagher, Asher Selznick, Carlos Prada, Yasser Al Shehab, Timothy Leroux, Moin Khan

**Affiliations:** 1 Bachelor of Health Sciences, Faculty of Health Sciences, McMaster University, Hamilton, ON, Canada; 2 Michael G. DeGroote School of Medicine, McMaster University, Hamilton, ON, Canada; 3 Division of Orthopaedic Surgery, Department of Surgery, McMaster University, Hamilton, ON, Canada; 4 Division of Orthopaedic Surgery, Department of Surgery, University of Toronto, Toronto, ON, Canada; 5 Department of Health Research Methods, Evidence, and Impact, McMaster University, Hamilton, ON, Canada

**Keywords:** Glenohumeral, cartilage, surgery, chondral defect, joint-preserving

## Abstract

**Background:**

Many joint-preserving surgical interventions for cartilage defects of the knee have been adapted for use in the shoulder; however, there still exists no clear consensus for treatment. Thus, the purpose of this systematic review was to evaluate the outcomes of different interventions in patients with focal chondral lesions of the glenohumeral joint.

**Methods:**

A literature search was conducted using PubMed, Embase, and Medline. Patients who underwent a joint-preserving surgical procedure to treat a focal chondral defect of the glenoid, humeral head or both were included. Patients treated for diffuse cartilage defects or with shoulder arthroplasty were excluded.

**Results:**

Ten studies were included, with follow-up data available for 194 shoulders. Eight joint-preserving procedures were evaluated, with microfracture being the most common. One study evaluating microfracture reported significant improvements in patient-reported outcomes at short-term and long-term follow-up compared to preoperative scores. Across all studies, 32 patients underwent subsequent shoulder surgery, with 22 being arthroplasties.

**Conclusions:**

We found improvements in patient-reported and functional outcomes across all studies. Although joint-preserving procedures have shown reasonable outcomes for focal chondral defects of the glenohumeral joint, long-term outcomes remain unknown, and the progression of osteoarthritis remains a concern. Higher quality evidence is required to make definitive recommendations.

**Level of Evidence:**

IV

## Introduction

Articular cartilage injuries vary widely, ranging from being asymptomatic to causing disabling pain.^1^ These types of injuries are most commonly seen in athletes, due to their highly active nature, and in older adults.^1,2^ Damage to articular cartilage is most common in weight-bearing joints such as the hip, knee, and ankle, but may also be present in non-weight-bearing joints such as the shoulder.^1,2^ In fact, symptomatic chondral lesions of the glenohumeral (GH) joint have been reported to have an incidence of 5–17%.^2–6^ A number of factors may contribute to the etiology of cartilage defects, including trauma, previous surgery, joint instability, osteonecrosis, osteochondritis dissecans, arthritis, and rotator cuff arthropathy.^3,7^

Currently, arthroplasty is the gold standard procedure to treat severe symptomatic diffuse GH cartilage defects.^8^ Although arthroplasty has been successful and is well accepted in treating diffuse cartilage defects in older, less active patients, joint-preserving procedures may be more beneficial for younger, more active patients, or for cases where the cartilage defect is focal.^3,8^ Notably, survival rates of total shoulder arthroplasty (TSA) in younger patients are not as good as in older, less active patients.^3^ With advancements in technology and surgical technique, joint-preserving procedures represent a promising avenue for the treatment of GH cartilage defects.^3^

A number of joint-preserving surgical interventions with established efficacy exist for cartilage defects of the knee, including arthroscopic debridement, microfracture, osteochondral autologous transplantation (OAT), osteochondral allograft transplantation (OCA), and autologous chondrocyte implantation (ACI), amongst others.^9,10^ Many of these procedures have been adapted for use in the GH joint; however, while published outcomes of individual procedures have demonstrated promising results, there is a scarcity of literature available assessing their long-term outcomes.^2^ The knee has received much more attention in this regard, and, as such, there exists no clear consensus amongst orthopedic surgeons on the indications, outcomes, and safety profile of the different surgical treatment techniques for chondral defects of the GH joint.^2,7,11^

Thus, the purpose of this systematic review is to compare the clinical outcomes and safety profile of different joint-preserving surgical interventions in patients with focal chondral lesions of the GH joint.

## Methods

### Search strategy

A comprehensive literature search was conducted on 22 October 2021, on three different databases: PubMed, Embase (via OVID), and Medline (via a web of science). Search strategies were tailored to their respective databases based on guidelines by Bramer et al.^12^ and Ceylan et al.^13^ Keywords used to perform the literature search included terms such as “cartilage,” “shoulder joint,” and variations of the term “cartilage lesions” such as “chondral defect,” “osteochondral lesion,” and so on (see Online Appendix 1).

### Eligibility criteria

Inclusion criteria included patients with focal chondral defects of the GH joint (humeral head, glenoid, or both) undergoing surgical intervention. There were no restrictions on age, gender, or ethnicity. Exclusion criteria included diffuse cartilage defects, non-English studies, animal or cadaveric studies, non-surgical studies, review articles, technique articles without outcomes, and case reports. This review focuses on joint-preserving procedures, thus shoulder arthroplasties (reverse shoulder arthroplasty (RSA), hemi-arthroplasty (HA), and TSA) were also excluded.

### Study screening

The screening process was completed by two independent reviewers using Covidence (Covidence systematic review software, Veritas Health Innovation, Melbourne, Australia). The selection of studies was performed in a stepwise manner, first by title and abstract, then full-text review. Conflicts were resolved by discussion and consensus among reviewers. The inter-rater reliability at each stage was measured using Cohen's kappa (*κ*) coefficients. The agreement was categorized a priori, as per Landis and Koch, with a value of 0.81–1.0 for near perfect agreement, 0.61–0.80 for substantial agreement, 0.41–0.60 for moderate agreement, and 0.21–0.40 for fair agreement.^14^

### Data collection

The data from the included studies were extracted independently by both reviewers using a data collection form on Microsoft Excel (version 15.32). Extracted data included study characteristics (year, study design, country, level of evidence, sample size, treatment type, follow-up), patient demographics (age, sex, lesion location, lesion size), outcome measures (patient-reported, functional, imaging results), and other information such as surgical details, complications and reoperations, study conclusions, and any additional comments.

### Quality assessment

The Methodological Index for Non-Randomized Studies (MINORS) was used to assess study quality, as this review only contains observational studies.^15^ The MINORS checklist for non-comparative studies comprises eight domains, each of which is assigned a score of 0, 1, or 2 with a maximum score of 16 points per study. MINORS scores for all studies are presented in Table 1. Each study was scored independently by two reviewers, and conflicts were resolved by discussion and consensus among reviewers. All studies were classified in their level of evidence according to the Oxford Center for Evidence-Based Medicine.^16^

**Table 1.** Study characteristics.

Study	Design	Country	MINORS score	Level of evidence	Sample size (final F/U)	Treatment type	Follow-up (range), months
Boehm et al. (2020)^17^	RCS	Germany	11 (69%)	IV	7	ACI	32 (22–58)
Buchmann et al. (2012)^18^	RCS	Germany	11 (69%)	IV	4	ACT-Cs	41.3 (11–71)
Cameron et al. (2002)^19^	RCS	United States	10 (63%)	IV	61	Arthroscopic debridement	34 (12–79)
Frank et al. (2010)^4^	RCS	United States	10 (63%)	IV	12 (13 shoulders)	Microfracture	27.8 (12.1–89.2)
Hünnebeck et al. (2017)^20^	RCS	Germany	9 (56%)	IV	32	Microfracture	105 (64–147)
Kircher et al. (2009)^21^	RCS	Germany	9 (56%)	IV	7	OAT	105 (91.2–117.6)
Millett et al. (2009)^22^	RCS	United States	10 (63%)	IV	30 (31 shoulders)	Microfracture	47 (25–128)
Riff et al. (2017)^5^	RCS	United States	10 (63%)	IV	20	OCA	66.5
Siebold et al. (2003)^23^	PCS	Germany	9 (56%)	IV	5	Microfracture + periosteal flap	25.8 (24–31)
Wang et al. (2018)^6^	RCS	United States	9 (56%)	IV	13 (14 shoulders)	Microfracture	122.4 (102–189.6)

Abbreviations: RCS: retrospective case series; PCS: prospective case series; ACI: autologous chondrocyte implantation; ACT-Cs: autologous chondrocyte transplantation with collagen membrane seeding; OAT: osteochondral autologous transplantation; OCA: osteochondral allograft transplantation.

## Results

### Characteristics of included studies

**Figure 1.**
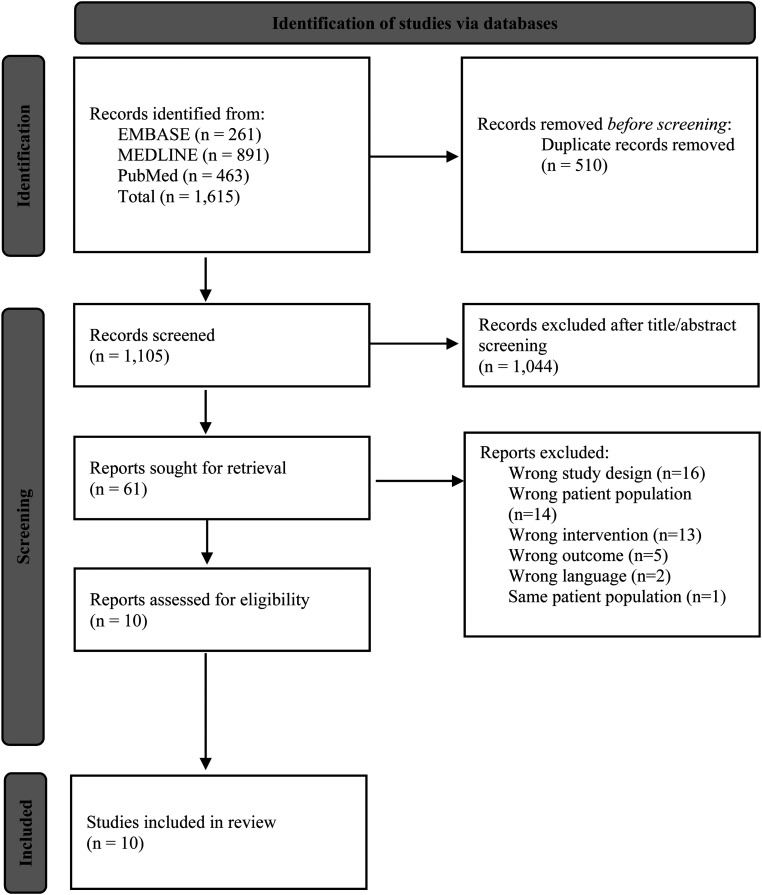
PRISMA study flow diagram.

Following duplicate removal, 1105 studies were assessed for eligibility. After title and abstract screening, 61 studies remained for full-text review. Following the full-text review, 10 studies were included in this review (Figure 1).^4–6,17–23^ The inter-rater reliability, measured using Cohen's kappa coefficients, yielded values of 0.35 (fair agreement) and 0.71 (substantial agreement) for the abstract and full-text screening, respectively. All studies presented level IV evidence, with MINORS scores ranging from 9 to 11 points (56–69%). The mean MINORS score across all studies is 10/16 (63%).

Of the 10 included studies, 9 (90%) were retrospective case series and 1 (10%) was a prospective case series. Follow-up data was available for a total of 194 shoulders in 191 patients. Microfracture was performed in 90 shoulders (46%),^4,6,20,22^ arthroscopic debridement in 61 shoulders with full-thickness isolated chondral defects (31%),^19^ OCA in 20 shoulders (10%),^5^ ACI in seven shoulders (4%),^17^ OAT in seven shoulders (4%),^21^ microfracture with periosteal flap in five shoulders (3%),^23^ and autologous chondrocyte transplantation with collagen membrane seeding (ACT-Cs) in four shoulders (2%).^18^ The average patient follow-up across all studies was 61 months (range: 25.8–122.4). Of the included studies, 5 (50%) originated from Germany^17,18,20,21,23^ and 5 (50%) from the United States.^4–6,19,22^ A detailed breakdown of study characteristics is presented in Table 1.

### Patient demographics and injury characteristics

Patient demographic information and injury characteristics are presented in Table 2. The mean age across all studies was 44 years (range: 16–74), with 129 patients (68%) being male. The location of the chondral lesions varied across as well as within studies and involved either the humeral head (HH), the glenoid, or both (GH). All but one study reported the lesion location, with a total of 39 bipolar lesions (20%) and 127 unipolar lesions (66%). The average lesion size was 3.8 cm^2^ at the HH and 1.6 cm^2^ at the glenoid. Only three studies (32 shoulders, 16%) reported the etiology of the cartilage defects, with intra-articular pain pump chondrolysis (*n* = 10) and anterior instability (*n* = 8, five recurrent and three traumatic) being the most common causes. For almost all patients, the initial indication for surgery was the treatment of a chondral defect, with additional concomitant procedures performed as needed.

**Table 2.** Patient demographics and injury characteristics.

Study	Age (range), years	Sex	Lesion location	Lesion size (range), cm^2^	Etiology of chondral defect
ACI					
Boehm et al. (2020)^17^	43 (18–55)	7 M, 0 F	7 H	H: 3.0 (2.3–4.5)	N/A
ACT-Cs					
Buchmann et al. (2012)^18^	29 (21–36)	4 M, 0 F	2 H, 1 G, 1 GH	H: 6.0	N/A
				G: 2.0	
Arthroscopic debridement					
Cameron et al. (2002)^19^	50 (21–73)	41 M, 20 F	19 H, 12 G, 30 GH	36 > 2.0 and 25 < 2.0	N/A
Microfacture					
Frank et al. (2010)^4^	37 (18–55)	7 M, 5 F	^a^10 H, 6 G, 1 GH	H: 5.1 (1.0–7.8)	N/A
				G: 1.7 (0.4–3.8)	
Hünnebeck et al. (2017)^20^	56 (37–74)	17 M, 15 F	N/A	N/A	N/A
Millett et al. (2009)^22^	43 (19–59)	25 M, 5 F	12 H, 13 G, 6 GH	N/A	N/A
Wang et al. (2018)^6^	36 (18–55)	6 M, 7 F	8 H, 5 G, 1 GH	H: 5.2 (4.0–7.8)	N/A
				G: 1.5 (1.0–3.8)	
Microfracture with periosteal flap					
Siebold et al. (2003)^23^	32 (16–56)	3 M, 2 F	5H	H: 3.1 (2.3–4.0)	1 idiopathic 1 hyperlaxity with posterior instability 1 recurrent anterior inferior instability 2 chondral damage caused by titanium anchor
OAT					
Kircher et al. (2009)^21^	45 (23–57)	6 M, 1 F	6 H, 1 G	H: 1.5 (1.1–2.5)	3 traumatic anterior instability 1 traumatic posterior instability 2 hyperlaxity 1 post-traumatic
				G: 1.3	
OCA					
Riff et al. (2017)^5^	25 (17–49)	13 M, 7 F	20 H	N/A	1 idiopathic 10 intra-articular pain pump chondrolysis 4 recurrent anterior instability 3 reverse Hill-Sachs 1 prominent suture anchors 1 radiofrequency chondrolysis (prior thermal capsulorrhaphy)

Abbreviations: M: male; F: female; H: humeral head; G: glenoid; GH: both articular surfaces; RCS: retrospective case series; PCS: prospective case series; ACI: autologous chondrocyte implantation; ACT-Cs: autologous chondrocyte transplantation with collagen membrane seeding; OAT: osteochondral autologous transplantation; OCA: osteochondral allograft transplantation.

^a^ Data for all patients, including those not available for follow-up; a breakdown of lesion location for only patients participating in follow-up is not available.

### Patient-reported and functional outcome measures

Outcome data are presented in Table 3. Patient-reported and functional outcomes were reported in all studies, but there was an important variability pertaining to the outcome measures reported. The most common outcome measures used were the American Shoulder and Elbow Surgeons Standardized Shoulder Assessment Form (ASES) in six studies (60%), the visual analog scale for pain (VAS) in five studies (50%), the Constant Shoulder Score (CSS) in five studies (50%), and the Simple Shoulder Test (SST) in three studies (30%). Of the different procedures included in this review, microfracture is the only procedure that was evaluated in more than one study (n = 4, 40%). Still, however, there was inconsistency pertaining to the outcome measures reported amongst these studies.

**Table 3.** Outcomes.

Study	Patient-reported	Functional	Imaging results
ACI			
Boehm et al. (2020)^17^	VAS Postop median: 0 at rest, 0 during exercise (range: 0–2)	ROM Free postop ROM No significant difference when compared to contralateral side	Radiologic evaluation 2 patients (29%) showed grade I secondary OA 32 months postop (range: 22–58)
	SSV Preop median: 60% (range: 30–60%) Postop median: 95% (range: 70–100%)		
	CSS Postop median: 95 pts (range: 80–100)		
	ASES Postop median: 97 pts (range: 90–100)		
ACT-Cs			
Buchmann et al. (2012)^18^	VAS Postop mean: 0.3	Rowe score Postop mean: 91.3 pts (range 75–100) No limitation in passive and active ROM compared to contralateral side	MRI scans 1 patient (25%) showed mild signs of OA postop
	Satisfaction scale 3 patients = very satisfied 1 patient = satisfied		
	CSS Postop mean: 83.3 pts (range 69–91)		
	ASES postop mean: 95.3 pts (range 83.3–100)		
Arthroscopic debridement			
Cameron et al. (2002)^19^	Pain score (at rest) Mean preop score: 5.0 pts Mean postop score: 1.9 pts	Functional score Mean preop score: 24 pts Mean postop score: 38.7 pts	N/A
	Pain score (light activities) Mean preop score: 6.9 pts Mean postop score: 3.1 pts		
	Pain score (strenuous activities) Mean preop score: 9.0 pts Mean postop score: 6.0 pts		
	Satisfaction score Mean preop score: 0.67 Mean postop score: 6.28		
	Mean improvement rate: 7.6		
Microfracture			
Frank et al. (2010)^4^	VAS Preop mean: 5.6 Postop mean: 1.9	N/A	N/A
	ASES Preop mean: 44.3 pts Postop mean: 86.3 pts		
	SST Preop mean: 5.7 Postop mean: 10.3		
Hünnebeck et al. (2017)^20^	DASH Operated shoulder mean: 12 pts Non-operated shoulder mean: 8 pts	N/A	X-ray findings 13 patients (57%) experienced progression of OA postop
	SSV Operated shoulder mean: 86 Non-operated shoulder mean: 88		
	CSS Operated shoulder mean: 74 pts Non-operated shoulder mean: 75 pts		
	Subjective evaluation At F/U, 13/27 patients reported no pain, and 12/27 patients reported moderate pain. Of these 12, 6/27 reported pain only at night and 3/27 only during rest 19/27 patients indicated that they were “satisfied” or “very satisfied” with the outcome of surgery		
Millett et al. (2009)^22^	Pain score Mean preop score: 3.8 pts Mean postop score: 1.6 pts	N/A	N/A
	Satisfaction score Mean postop score: 7.6		
	ASES Preop mean: 60 pts (range: 20–80) Postop mean: 80 pts (45–100)		
Wang et al. (2018)^6^	Satisfaction score Mean postop score: 9.5 (range: 7–10)	N/A	N/A
	VAS Preop mean: 5.9 (range: 1–9) Postop mean (short-term): 1.8 (range: 0–7) Postop mean (long-term): 1.5 (range: 0–4)		
	ASES Preop mean: 43.6 (range: 25–85) Postop mean (short-term): 86.3 (range: 45–100) Postop mean (long-term): 88.1 (range: 73.3–100)		
	SST Preop mean: 5.3 (range: 1–11) Postop mean (short-term): 10.3 (range: 6–12) Postop mean (long-term): 10.4 (range: 5–12)		
Microfracture with periosteal flap			
Siebold et al. (2003)^23^	CSS Preop mean: 43.4% (range: 28–58) Postop mean: 81.8% (range: 59–94)	N/A	Radiologic findings 2 patients (40%) with preop OA deteriorated postop
OAT			
Kircher et al. (2009)^21^	CSS Preop mean: 76.2 pts (range: 65.9–89.6) Postop mean (first F/U): 89.6 pts (range: 83.4–95.4) Postop mean (final F/U): 90.9 pts (range: 80–97)	N/A	Radiologic findings All patients experienced progression of OA at first F/U (mean 32 months) 2 patients (29%) showed further progression of OA at final F/U (mean 8.8 years)
OCA			
Riff et al. (2017)^5^	VAS Preop mean: 5.8 Postop mean: 1.9	N/A	N/A
	SF-12 Preop mean: 37.8 Postop mean: 48.7		
	ASES Preop mean: 40.8 Postop mean: 75.8		
	SST Preop mean: 31.9 Postop mean: 76.8		

Abbreviations: VAS: Visual Analog Scale; DASH: Disabilities of the Arm, Shoulder and Hand; SSV: Subjective Shoulder Value; CSS: Constant Shoulder Score; ASES: American Shoulder and Elbow Surgeons Standardized Shoulder Assessment Form; SST: Simple Shoulder Test; SF-12: 12-Item Short Form Survey, ROM: Range of Motion; OA: osteoarthritis.

*Pain score: 10-point max.

*Satisfaction score: 10-point max.

*Improvement rate: 10-point max.

*Functional score: 60-point max.

Wang et al.^6^ and Kircher et al.^21^ were the only studies that reported values for outcomes at short-term (mean 2.3 years for both) as well as long-term (mean 10.2 and 8.8 years, respectively) time points. For Wang et al.,^6^ pairwise comparisons revealed that VAS, ASES, and SST scores were significantly improved at both short-term and long-term follow-up as compared with preoperative scores. However, there was no significant difference in VAS, ASES, or SST scores between short- and long-term follow-up.^6^ For Kircher et al.,^21^ CSS scores improved significantly (*P* = 0.018), with an increase of 13 points between the preoperative and short-term time points, and an increase of 15 points between the preoperative and long-term time points.

Three studies (30%) compared outcomes between patients who did and did not undergo any concomitant procedures. Cameron et al.^19^ noted that no statistical difference was found in final pain scores, functional scores, improvement rate, satisfaction scores, or duration of pain relief between both patient subgroups (with and without concomitant procedures) (*P* = 0.5). Similarly, Wang et al.^6^ found no significant difference in long-term patient-reported outcome scores (VAS, *P* = 0.630; SST, *P* = 0.714; ASES, *P* = 0.714) or progression to failure (*P* = 0.999) between subgroups. Frank et al.^4^ also noted postoperative improvements in VAS, SST, and ASES scores in both subgroups.

### Imaging results

Five studies (50%) reported imaging results, which were generally used to assess the progression of osteoarthritis (OA) in the operated shoulder.^17,18,20,21,23^ Siebold et al.^23^ reported that, of the patients included in their study (n = 5), two patients (40%) with preoperative OA deteriorated postoperatively. The other three patients (60%) had no preoperative signs of OA and showed no signs of change at an average of 26 months postoperatively.^23^ Kircher et al.^21^ reported that all patients (n = 7) experienced progression of OA at first follow-up (mean 32 months), but only three patients (43%) showed preoperative signs of OA. Two patients (29%) showed further progression of OA at final follow-up (mean 8.8 years), both of which had preoperative signs of OA.^21^ Hünnebeck et al.^20^ reported a statistically significant increase in radiological signs of OA postoperatively, with patients with preoperative signs of OA progressing to higher stages (according to the Samilson and Prieto classification) than those without preoperative signs (*P* = 0.013).^24^

Of these five studies, three reported defect filling rates assessed by follow-up MRI evaluations. Boehm et al.,^17^ evaluating ACI, reported that complete defect coverage was achieved in three patients (43%), but that a residual defect was present in one patient (14%). Siebold et al.,^23^ evaluating microfracture with periosteal flap, reported that in all patients, the area of the chondral defect was covered with a thin layer of regeneration cartilage tissue. Kircher et al.,^21^ evaluating OAT, reported that six out of seven patients (86%) presented a congruent joint surface on the final MR scan and all cylinders showed full integration with the surrounding bone.

### Complications and reoperations

Complications were only reported in two types of procedures: ACI and OAT.^17,21^ Complications associated with the ACI procedure were encountered in one patient (14%), who had postoperative stiffness of the GH joint as a result of adhesive capsulitis.^17^ Regarding the OAT procedure, there were no complications at the shoulder, but one patient (14%) developed donor site morbidity at the knee, characterized by persistent pain and recurrent effusions.^21^

Reoperations were reported in almost all studies (n = 7, 70%). Amongst the studies evaluating microfracture, 18 patients (20%) required reoperations, of which 12 (67%) were shoulder arthroplasties (five HAs, six TSAs, one RSA).^4,6,20,22^ Hünnebeck et al.^20^ reported that the mean time until subsequent shoulder surgery (three HAs and two TSAs) was 47 months (range: 5–79) after the index microfracture procedure. In the study evaluating arthroscopic debridement, six patients (10%) ultimately required a shoulder arthroplasty (one HA, five TSAs) at an average of 16.3 months after the index procedure.^19^ In the study evaluating OCA, four patients (20%) ultimately required a TSA at an average of 25 months after the index procedure.^5^ For ACI, one patient (14%) underwent 270° capsular release and a capsulectomy at 12 months after the index procedure.^17^ Details pertaining to complications and reoperations are presented in Table 4.

**Table 4.** Complications and reoperations.

Study	Complications	Reoperations
ACI		
Boehm et al. (2020)^17^	1 GH postop stiffness secondary to adhesive capsulitis	1 patient (14%) underwent 270^o^ capsular release, as well as a ventral and inferior capsulectomy at 12 months of F/U
ACT-Cs		
Buchmann et al. (2012)^18^	N/A	N/A
Arthroscopic debridement		
Cameron et al. (2002)^19^	N/A	6 patients (9.8%) required subsequent shoulder surgery at a mean of 16.3 months (range: 2–48) after the index procedure 1 HA 5 TSAs
Microfacture		
Frank et al. (2010)^4^	N/A	3 patients (23%) required subsequent shoulder surgery 1 arthroscopic debridement and capsular release 1 biological resurfacing 1 HA
Hünnebeck et al. (2017)^20^	N/A	5 patients (16%) required subsequent shoulder surgery at a mean of 47 months (range: 5–79) after the index procedure 3 HAs 2 TSAs
Millett et al. (2009)^22^	N/A	6 patients (19%) required subsequent shoulder surgery 3 TSAs 1 arthroscopic biceps release 1 RCR with thermal capsulorrhaphy 1 type of surgery unknown
Wang et al. (2018)^6^	N/A	4 patients (29%) required subsequent surgeries 1 HA (0.2), revision shoulder arthroplasty (8.8), capsular release (10.7)^a^ 1 distal tibia allograft (1.4), debridement with subacromial decompression and distal clavicle excision (5.7), pectoralis major transfer (6.6), RSA (7.3)^a^ 1 SLAP repair, posterior labral repair, anterior stabilization, HH chondroplasty (2.8), HH debridement with glenoid microfracture and capsular release (9.5), TSA (9.6)^a^ 1 debridement with synovectomy, subacromial decompression, and distal clavicle excision (2.7)^a^
Microfracture with periosteal flap		
Siebold et al. (2003)^23^	N/A	N/A
OAT		
Kircher et al. (2009)^21^	1 patient had donor site morbidity at the knee (persistent pain and recurrent effusions) but no complications at the shoulder	N/A
OCA		
Riff et al. (2017)^5^	N/A	4 patients (20%) required subsequent shoulder surgery at a mean of 25 months after the index procedure 4 TSAs

Abbreviations: GH: glenohumeral; TSA: total shoulder arthroplasty; HA: hemiarthroplasty; RCR: rotator cuff repair; RSA: reverse shoulder arthroplasty; RCS: retrospective case series; PCS: prospective case series; ACI: autologous chondrocyte implantation; ACT-Cs: autologous chondrocyte transplantation with collagen membrane seeding; OAT: osteochondral autologous transplantation; OCA: osteochondral allograft transplantation.

^a^ The numbers in brackets represent the number of years that the subsequent procedure was performed after the original microfracture procedure.

## Discussion

This review found improvements in clinical outcomes across all studies, with most studies reporting significant improvements in pain relief and shoulder function with surgical management of isolated chondral defects at an average of 61 months postoperatively. All studies evaluating microfracture found that this technique provides significant improvement in pain relief and shoulder function in patients with isolated full-thickness chondral injuries.^4,6,20,22^ Findings by Wang et al.^6^ suggest that the long-term clinical outcomes of microfracture as a treatment for full-thickness cartilage defects of the GH joint are durable and similar to previously reported short-term outcomes. This study reported statistically significantly different VAS, ASES, and SST scores between the preoperative and short- and long-term follow-up time points (mean 2.3 and 10.2 years, respectively), but no significant difference in these scores between short- and long-term follow-up.^6^ Interestingly, no decay was seen between short- and long-term outcomes, suggesting that the improvements were sustained over time. Kircher et al.,^21^ evaluating OAT, also noted that the increase in CSS scores between the preoperative and short-term time points was statistically significant (*P* = 0.018), but not between the short-term and long-term time points (*P* = 0.612), suggesting no decay in the results up to an average of 8.8 years.

Another important finding is that patients with unipolar lesions improve significantly better than patients with bipolar lesions. Millett et al.^22^ noted that the greatest improvement was seen in patients in whom just the HH was treated, with an increase of 32 points in the ASES score at an average of 47 months postoperatively. This increase largely surpasses that of the reported MCID of 15 points for the ASES score.^25,26^ Hünnebeck et al.^20^ noted similar results, stating that patients with bipolar lesions showed statistically significantly poorer SSV scores (*P* = 0.039) than patients with unipolar lesions. While Riff et al.^5^ evaluated a different procedure, they also noted favorable results in patients with isolated defects of the HH, at an average follow-up of 5.5 years, reporting a mean increase of 35 points in the ASES scores, surpassing the MCID. In patients with bipolar lesions or etiology secondary to an intra-articular pain pump, OCA transplantation has an increased risk of failure and less favorable subjective outcomes. Among the 20 patients who underwent the OCA procedure in this study, 10 (50%) had an etiology of chondrolysis secondary to continuous intra-articular infusion of local anesthetic via a pain pump. Of the seven patients who were dissatisfied with the OCA procedure, six of them had a history of an intra-articular pain pump, and all six of these patients required a subsequent operation (including four who ultimately required TSA).

In addition, Cameron et al.,^19^ evaluating arthroscopic debridement, reported that return of pain and ultimate failure were significantly related to lesions greater than 2 cm^2^ (*P* < 0.003), and that the mean time until maximum pain relief was achieved was 11 weeks after surgery. Amongst patients who had no pain relief (*n* = 7) or whose pain returned (*n* = 19), 22 patients (85%) had lesions greater than 2 cm^2^ (*P* < 0.003). Importantly, the authors noted that the mean age of those who underwent arthroplasty was 60 years, and all patients had lesions that were greater than 2 cm^2^.

The reported rate of surgical complications was very low, appearing only in two patients (1%): one treated with ACI and one treated with OAT. However, the need for subsequent procedures is a significant concern, as it was reported in almost all studies. Across all studies, a total of 29 patients (15%) underwent subsequent shoulder surgery, with 22 (76%) of these reoperations being shoulder arthroplasties (15 TSAs, six HAs, one RSA). Findings by Wang et al.^6^ demonstrate that, although GH microfracture can provide lasting improvement in some patients, it is associated with a reoperation rate of 29%, with 21% of patients requiring conversion to arthroplasty less than 10 years after the index procedure. The overall long-term success rate of GH microfracture in this study is 66.7%, with three structural failures (defined as conversion to arthroplasty or biological resurfacing), and two clinical failures (defined by patient dissatisfaction).^6^ Across all studies, a total of 22 patients (11%) ultimately required shoulder arthroplasties (15 TSAs, six HAs, one RSA).

In most studies that reported imaging results, the progression of GH OA was commonly reported. Overall, patients without any preoperative signs of OA generally had better outcomes. However, Hünnebeck et al.^20^ concluded that, even though microfracture does not prevent the radiographic progression of OA, it might be worth considering as part of a treatment regimen for younger patients who may not yet be treated with arthroplasty. Kircher et al.^21^ noted that all patients experienced progression of OA at the first follow-up, and two patients (29%) showed further progression of OA at the final follow-up. However, the progression of OA was not matched by any functional restriction, pain, or loss of patient satisfaction. At this time, it is difficult to determine whether the arthritic changes at the shoulder were due to the procedure, or reflected the natural course of the underlying disease.

It remains unclear whether there is an association between the etiology of the defect and the postoperative outcomes. Due to the lack of long-term follow-up time points available, it also remains unclear how long these procedures will successfully provide symptomatic relief and delay subsequent procedures. Moreover, concomitant procedures were performed for a large number of patients. It is nearly impossible to determine whether the chondral defects were responsible for the preoperative symptoms in certain patients and if the treatment for these defects were wholly or partly responsible for the clinical improvements.^4^ Nonetheless, all studies concluded that their respective procedures constitute a viable treatment option for isolated chondral lesions of the shoulder joint.

### Applicability of evidence

This systematic review included 10 studies evaluating a total of 194 shoulders in 191 patients. Microfracture is the only surgical technique that was appraised in more than one study included in this review. As such, it would be premature to draw any solid conclusions pertaining to superiority based on the available evidence. Moreover, it is difficult to assess the consistency of the results due to the heterogeneity of the outcome measures reported, the different nature of the chondral lesions (size and location), and the varying follow-up time points. Amongst the studies that did report the same outcome measures, some studies reported postoperative but not preoperative measures, making it difficult to assess relative improvement. A systematic review by Fiegen et al.^27^ published in 2019 presented similar findings as those in the current review. The current review, however, excluded case reports, which may increase the strength of the evidence. Additionally, this review evaluated a larger sample size (194 shoulders vs 100 shoulders) and more types of procedures (inclusion of arthroscopic debridement and ACI) than the previous review.

### Limitations and future directions

There are several limitations in this systematic review. First, this review consists only of studies with level IV evidence. The nature of the interventions being evaluated and the relatively low incidence of these injuries render it more difficult to conduct studies with a prospective design, which could provide answers from a higher level of evidence. The studies also lack blinding, randomization, and comparative control groups. Second, the included studies generally had a small sample size and variability in the condition being treated, which may decrease the generalizability of the results. Looking at the geographic distribution of the included studies, data only comes from two countries (Germany and the United States), which may also decrease the generalizability of the results. Moreover, post-op assessments were limited by the nature of the imaging technique, as MRI findings are difficult to assess the quality of the chondral repair. Second-look arthroscopy, which would provide a more comprehensive assessment, is not always feasible. More studies assessing long-term data with larger patient populations and a multi-centric design are required to determine which joint-preserving surgical technique should be the standard procedure for the treatment of GH cartilage defects.

## Conclusions

There exists a variety of surgical techniques for the management of chondral defects of the GH joint. Based on our findings, microfracture appears to be the most commonly used intervention for the treatment of this condition. This is likely due to the fact that this procedure is relatively easy to perform, cost-effective, and has low patient morbidity.^28^ Although there is still no clear consensus as to which joint-preserving approach should be the standard of care, the findings of this systematic review demonstrate improvements in patient-reported and functional outcomes across all studies, with most patients experiencing pain relief. However, the progression of OA and the substantial need for reoperations remains a concern that should be considered and discussed with this patient population. While the procedures evaluated in this review may constitute viable treatment options for chondral lesions of the shoulder joint, higher-quality evidence is required to make definitive recommendations.

## Supplemental Material

sj-docx-1-sel-10.1177_17585732221142610 - Supplemental material for Surgical treatment options for articular cartilage defects of the glenohumeral joint: A systematic review Click here for additional data file. Supplemental material, sj-docx-1-sel-10.1177_17585732221142610 for Surgical treatment options for articular cartilage defects of the glenohumeral joint: A systematic review by Danielle Dagher, Asher Selznick, Carlos Prada, Yasser Al Shehab, Timothy Leroux and Moin Khan in Shoulder & Elbow
