# Supplementary material for: Surgical treatment options for articular cartilage defects of the glenohumeral joint: A systematic review
Source: Shoulder Elbow. 2022 Dec 14;15(6):580–92. doi: 10.1177/17585732221142610 (PMC10656978; doi:10.1177/17585732221142610)
Supplement: sj-docx-1-sel-10.1177_17585732221142610 - Supplemental material for Surgical treatment options for articular cartilage defects of the glenohumeral joint: A systematic review [file sj-docx-1-sel-10.1177_17585732221142610.docx]

Appendix 1. Search Strategies

**Pubmed: 463 results**

1. "Cartilage, Articular"[Mesh]

2. "Shoulder Joint"[Mesh]

3. 1 AND 2

**Embase (via OVID): 261 results**

1. cartilage/

2. shoulder joint.mp. or shoulder/

3. 1 AND 2

**Medline (via Web of Science): 891 results**

1. (cartilage lesion OR cartilage lesions OR cartilage defect OR cartilage defects OR osteochondral lesion OR osteochondral lesions OR osteochondral defect OR osteochondral defects OR chondral lesion OR chondral lesions OR chondral defect OR chondral defects OR cartilage OR chondral)

2. Shoulder Joint (MeSH Heading)

3. 1 AND 2
